# Autoimmune Abnormalities of Postpartum Thyroid Diseases

**DOI:** 10.3389/fendo.2017.00166

**Published:** 2017-07-13

**Authors:** Flavia Di Bari, Roberta Granese, Maria Le Donne, Roberto Vita, Salvatore Benvenga

**Affiliations:** ^1^Department of Clinical and Experimental Medicine, University of Messina, Messina, Italy; ^2^Department of Obstetrics and Gynecology, University Hospital “G. Martino”, Messina, Italy; ^3^Master Program on Childhood, Adolescent and Women’s Endocrine Health, University of Messina, Messina, Italy; ^4^Interdepartmental Program of Molecular & Clinical Endocrinology, and Women’s Endocrine Health, University Hospital “G. Martino”, Messina, Italy

**Keywords:** postpartum thyroiditis, thyroid autoimmunity, non-autoimmune thyroiditis, Graves’ disease, thyroid autoantibodies

## Abstract

The year following parturition is a critical time for the *de novo* appearance or exacerbation of autoimmune diseases, including autoimmune thyroid disease. The vast majority of postpartum thyroid disease consists of postpartum thyroiditis (PPT) and the minority by Graves’ disease and non-autoimmune thyroiditis. PPT has a worldwide prevalence ranging from 1 to 22% and averaging 5% based on a review published in 2012. Several factors confer risk for the development of PPT. Typically, the clinical course of PPT is characterized by three phases: thyrotoxic, hypothyroid, and euthyroid phase. Approximately half of PPT women will have permanent hypothyroidism. The best humoral marker for predictivity, already during the first trimester of gestation, is considered positivity for thyroperoxidase autoantibodies (TPOAb), though only one-third to half of such TPOAb-positive pregnant women will develop PPT. Nutraceuticals (such as selenium) or omega-3-fatty acid supplements seem to have a role in prevention of PPT. In a recent study on pregnant women with stable dietary habits, we found that the fish consumers had lower rates of positivity (and lower serum levels) of both TPOAb and thyroglobulin Ab compared to meat eaters. Finally, we remind the reader of other diseases that can be observed in the postpartum period, either autoimmune or non-autoimmune, thyroid or non-thyroid.

## Introduction

The postpartum period, especially its first year, is a critical time for the onset, exacerbation, or relapse of autoimmune diseases. The literature on this topic is relatively scarce, as a PubMed search run on February, 2017 by using the string “postpartum and autoimmunity” yielded only 139 articles. Before discussing of postpartum thyroid diseases (PPTDs) and especially of postpartum thyroiditis (PPT), we would like to remind the existence of autoimmune endocrine diseases involving glands other than thyroid (1–20) as well as of thyroid diseases of non-autoimmune nature (21–27), as summarized in Table 1.

**Table 1:** Endocrinopathies in the postpartum period.

Endocrinopathy	Comments
Postpartum Addison’s disease (1–7)	It is a rare disease. Its diagnosis can be overlooked during pregnancy and after parturition. Indeed, fatigue, anorexia, vomiting, and hyperpigmentation can be easily confused with similar symptoms that occur frequently during gestation and/or postpartum. Because unrecognized, acute, and frequently fatal addisonian crises may occur in the postpartum period
Hypopituitarism [Postpartum lymphocytic hypophysitis (PPLH) and Sheehan’s syndrome] (8–20)	PPHL or autoimmune hypophysitis is mostly observed in women during pregnancy or after delivery, though it may also occur in males and children. PPLH is frequently associated with autoimmune diseases, particularly with Hashimoto’s thyroiditis
	Either PPLH or the non-autoimmune postpartum pituitary gland ischemic necrosis (Sheehan’s syndrome) can be associated with postpartum thyroiditis (PPT)
Non-autoimmune thyroiditis (21–27)	This form of thyroiditis is far less frequent than PPT
	Infective forms of thyroiditis with subacute or acute course have been reported in the postpartum setting caused by *Brucella melitensis* or *Mycobacterium tuberculosis*. The symptoms reproduce a thyrotoxic picture (nervousness, palpitations, and loss of weight) with moderately painful goiter and fever (37.5°C). It is important to emphasize that in some cases inflammatory changes seen in subacute thyroiditis can obscure sonographic evidence of underlying papillary thyroid cancer. Also, a clinical picture of painful thyroid enlargement, even with fever, and local mechanical complications can be due to intrathyroid hemorrhages

## Postpartum Thyroid Autoimmunity

### Postpartum Graves’ Disease (GD)

As well known, GD is an autoimmune disorder characterized by hyperthyroidism, with or without associated ophthalmopathy. Its pathogenesis is related to loss of tolerance to autoantigen thyroid-stimulating hormone receptor that leads to the infiltration of the gland.

Studies indicate that new-onset autoimmune thyroid disease (AITD) “occurs in up to 10% of all women in the postpartum period and that up to 60% of GD patients in the reproductive years give a history of postpartum onset” (28).

Two contemporary Canadian studies in the same province (Ontario) but in different areas and on different categories of women found different frequencies of PPT and postpartum GD (29, 30). The Toronto area study on 1,372 unselected women found that 78 (5.7%) had PPT and 3 (0.22%) GD; in addition, 1 other woman (0.07%) had postpartum thyrotoxicosis due to toxic nodular goiter (29). Thus, the PPT to postpartum GD ratio was 78:3 (26:1). Instead, in 40 Canadian women with type 1 diabetes mellitus (DM1) residing in the Hamilton area, the ratio was 9:1, since PPTD consisted of PPT in 9 patients (22.5%) and postpartum GD in 1 patient (2.5%) (30). The ratio between PPT and postpartum GD can be inferred from additional studies. In one Iranian investigation on 1,040 pregnant women (31), 119 had PPT (11.4%) and only 1 GD (1%), with a ratio of 119:1. Instead, a Spanish study on 641 pregnant women (32), not all of whom sampled at all-time points throughout 12 months postpartum, found that 45 developed PPT [incidence rate 7.8%; confidence interval (CI) 5.6–10%], 8 developed GD (incidence rate 1.5%; CI 0.5–2.5%) and 3 developed non-palpable toxic thyroid adenoma-associated hyperthyroidism (incidence rate 0.5%; CI 0–1.5%). Thus, the ratio between PPT and postpartum GD was 6:1. Incidentally, this 8:3 (2.7:1) ratio between postpartum GD and postpartum toxic adenoma matches the 3:1 ratio of the aforementioned Canadian study (29). According to Japanese authors (33), the frequency of postpartum GD in the general population is estimated at around 0.5%, that is, 1 in 200 postpartum women.

In two retrospective Italian studies (34, 35), the postpartum period was a risk factor for relapse(s) of GD, not for the onset. Instead, an earlier Swedish study concluded for a risky role of the postpartum period in the onset of GD (36). In this study (36), 93 consecutive women with GD aged 20–40 years were examined for a possible relation between onset of GD and previous pregnancy. An increased relative risk of developing GD within 1 year following delivery was found (RR = 6.5, CI 3.8–11.0). After excluding the nulliparous women, almost two out of three women who developed GD in the childbearing age of 20–35 years had a postpartum onset, suggesting an important role of immunomodulatory events following delivery for the development of this disease in young women. A similar relative risk (that is, RR = 5.6) was reported for American women aged 35–39 years (37). This is retrospective study on 152 consecutive women, aged 18–39 years when diagnosed with GD (37). The authors found that, in parous women, 45% were diagnosed with GD in the postpartum period and 55% had an onset in subsequent years. The risk of developing post-pregnancy GD was the greatest in the age band 35–39 years, with 56% of them developing GD compared to 42% of nulliparous women (37). In a Japanese retrospective study on 289 consecutive women with GD, 92 were of childbearing age (20–39 years) and had one or more deliveries (38). At least 37 patients had evident postpartum onset of the disease. Thus, at least 40% of GD women aged 20–39 years developed their disease during the postpartum period (38). In another study, in women diagnosed with GD during the ages of 20–35 years, 66% had a postpartum onset, and women with a previous history of GD relapsed frequently in the postpartum period (39).

In a French study (40), 98 patients with GD were compared to 95 patients with Hashimoto’s thyroiditis (HT) and to 97 patients with benign thyroid nodules (control group) in order to evaluate the triggering role of pregnancy and other major stressors in the occurrence of AITDs. A stress factor was encountered in 11% of GD women and 6% of HT or thyroid nodularity women, an insignificant difference. Instead, in women of childbearing age, GD after a pregnancy occurred more frequently than HT or benign thyroid nodules (25 vs. 10 or 13%, *p* < 0.05). The author concluded that the role of stressors, if any, in triggering GD seems to be weak and dubious compared to the role of pregnancy and postpartum.

Concerning possible prevention of GD relapse in the postpartum, continuing antithyroid drugs therapy throughout pregnancy prevents recurrence of GD without neonatal hypothyroidism or malformations (41). However, the use of methimazole is related to its potential teratogenic effects, especially in the first trimester of pregnancy (42).

Concerning medical therapy of GD and in view of the reference made to the omega-3 fatty acids-rich fish in the next heading, it is noteworthy mentioning the following case (43). A professor of physiology self-reported her GD developed 4 months postpartum with a TSH normalization, within 8 weeks of beginning flaxseed oil supplement (5–1,000 mg tablets twice a day). Flaxseed oil is over 50% omega-3 fatty acids (mainly alpha-linolenic acid), but it also contains about 15% omega-6 fatty acids (mainly linoleic acid) (43).

It is noteworthy that GD and PPT may affect the same woman, at different times. A clear case of PPT (in the form of transient thyrotoxicosis) in a young woman in whom GD had appeared 6 years earlier was reported (44). Shortly prior to becoming pregnant, an increase in thyroperoxidase autoantibodies (TPOAb) was observed in this woman. In another Japanese woman with thyroid hemiagenesis, GD was present in the pregnancy that preceded the postpartum period during which PPT appeared (45). Other case reports of PPT following GD have appeared in the literature (46, 47), one being noteworthy because of the sequence onset of GD → PPT → relapse of GD (47). The chronological sequence of PPT preceding GD was described in three young Caucasian women (48, 49). Based on the different pattern of radioiodine thyroid uptake, rate of positivity and mean levels of TSH-binding inhibiting antibodies, and evolution of hyperthyroidism in almost 100 women with GD followed up in the postpartum period, it was concluded that PPTD develops frequently in the postpartum period of patients with GD (50).

Finally, concurrent Sheehan’s syndrome and GD in the postpartum has been reported (51). In this case report, GD appeared first and Sheehan’s syndrome later.

### Postpartum Thyroiditis

In this minireview, we will focus on the endocrine and autoimmune side of PPT, with no reference to the neuropsychological disturbances, particularly the association with postpartum depression (52, 53). Neuropsychological disturbances, such as acute psychosis, can also occur in association with postpartum GD (54). Very recently, we have reviewed the postpartum mood disorders (55).

Postpartum thyroiditis is a thyroid dysfunction, characterized by lymphocytic infiltration of the gland, which appears in the first postpartum year in women who were euthyroid prior to pregnancy (42, 53). The just released guidelines of the American Thyroid Association (42) endorse the original definition by Amino et al. (56), namely PPT is “the occurrence of thyroid dysfunction, excluding GD, in the first postpartum year in women who were euthyroid prior to pregnancy.”

#### Epidemiology and Risk Factors

Based on a review published in 2012 (53), PPT has a worldwide prevalence of approximately 5%, but ranging from 1 to 22%. Because of its autoimmune nature, other autoimmune disorders may appear years after PPT. In one study, 40 women (mean age 36 years) with documented PPT 5 years earlier and 30 age-matched healthy women who all had undergone normal delivery an average of 5 years previously, were investigated (57). Women with previous PPT had symptoms of dry eyes, caries, arthralgias, swollen joints, and fatigue significantly more often than control group (*p* < 0.05). One-third (34%) of the women were anti-SSB positive and 46% were anti-SSA positive at follow-up. Furthermore, 15/35 women with a history of PPT had objectively reduced tear and/or saliva secretion; 5/24 investigated women had keratoconjunctivitis sicca, and 2/7 salivary gland biopsies showed chronic lymphocytic sialadenitis. Three women (8.6%) had both xerophthalmia and xerostomia. The authors concluded that features of Sjögren’s syndrome are frequent in young women with previous PPT (57).

Several factors confer risk for the development of PPT, a major one being serum positivity for thyroid autoantibodies (TAb) during gestation (53). Furthermore, based on a systematic review (58), the risk for PPT conferred by positivity for TAb during gestation is much greater than the risk for other outcomes. Indeed, compared with women who are TAb negative, odds ratio (OR) for maternal PPT was 11.5, greater than the OR for miscarriage (3.73), recurrent miscarriage (2.3), preterm birth (1.9), or unexplained subfertility (1.5) (58). Paralleling GD, parturition can be a risk factor for autoimmune thyroiditis (AIT) (59). Women with at least one pregnancy had increased likelihood for AIT (OR 4.6, *p* < 0.05) compared to women who have never been pregnant. Similar results were observed using hypoechogenic thyroid pattern (OR 1.7, *p* < 0.05) and positive TPOAb levels (OR 1.8, *p* = 0.05) as separate dependent variables or using number of births as alternate independent variable. Furthermore, immune rebound after parturition may cause not only AITD, including PPT, but other autoimmune diseases: postpartum renal failure or postdelivery hemolytic–uremic syndrome, postpartum idiopathic polymyositis, postpartum syndrome with antiphospholipid antibodies, and postpartum autoimmune myocarditis (60).

Women with other autoimmune disorders as systemic lupus erythematosus (61), chronic viral hepatitis (62), DM1 (63–66), multiple sclerosis (67), and antipituitary antibodies positivity (19) have an increased risk of PPT (42) (Table 2). Other conditions that can predispose to develop PPT, such as gestational diabetes (68), are summarized in Table 2. Also, PPT may develop years after irradiation of the neck for lymphoma (69).

**Table 2:** Frequency of postpartum thyroiditis (PPT) in women with the indicated disease or condition.

Reference	Disease/condition	Population studied	Frequency of PPT	Comments
Stagnaro-Green et al. (61)	Systemic lupus erythematosus (SLE)	63 pregnant women with SLE	14%	
Elefsiniotis et al. (62)	Chronic viral hepatitis (HCV and HBV)	21 women with chronic HCV infection and 74 women with chronic HBV, of whom 16 and 64 finally included in the study	Four of 16 chronic HCV-infected women (25%) and none of 64 chronic HBV infected women developed PPT	All chronic HBV-infected women had never been treated before whereas 3 of 16 chronic HCV-infected women had been treated in the past with pegylated-interferon alpha plus ribavirin
Bech et al. (63)	Type 1 diabetes mellitus (DM1)	85 pregnant women with DM1	10.5%	
Gallas et al. (64)	DM1	126 pregnant women with DM1	15.9%	Patients with postpartum thyroid disease (PPTD) were slightly older than those without PPTD and the prevalence of TPO-Ab was higher in these women
Alvarez-Marfany et al. (65)	DM1	41 pregnant women with DM1	25%	25% was threefold greater in a non-diabetic population studied by the same group of authors. Forty-three percent of the women (3/7) who developed PPTD required treatment in the immediate postpartum period and at long-term follow-up (permanent hypothyroidism)
Gerstein (30)	DM1	51 pregnant women with DM1, 40 of whom completed follow-up	22%	Postpartum thyroid dysfunction occurred in 10 of 40 patients (25%; 95% confidence interval, 12.7–41.2%); PPT developed in 9 patients (22.5%) and postpartum Graves’ disease developed in 1 patient (2.5%)
Triggiani et al. (66)	DM1	15 DM1 pregnant women vs 10 age-matched healthy controls	13.3% in DM1 women vs 20% in healthy controls	
Jalkanen et al. (67)	Multiple sclerosis (MS)	46 MS pregnant women vs 35 age-matched healthy controls	3.4% in MS women vs 2.9% in healthy controls	PPT rate in MS and controls was similar (3.4 and 2.9%) despite the fact that the rate of elevated serum levels for thyroid autoantibodies (TAb) at 6 months postpartum was sixfold greater in MS (35.3 vs 5.7%)
Komatsu et al. (69)	Irradiation of the neck	Case of a 30-year-old Japanese woman		Irradiation therapy to the neck for malignant lymphoma 9 years earlier
Paragliola et al. (70) Taniyama, et al. (71)	Thyroid hormone resistance (RTH) syndrome	Case of a 30-year-old Italian woman Case of a 44-year-old Japanese woman		RTH was due to a mutation of thyroid hormone receptor β, but occurring at different codons in these two women
Galanti et al. (73)	Smoking cigarettes	874,507 parous women smoking during pregnancy	Thyroiditis within 6 months from childbirth was positively associated with smoking (adjusted HR = 1.88)	Smoking may increase the risk of thyroiditis occurring in the postpartum
Balázs and Farid (74)	Smoking cigarettes	22 pregnant women with previous PPT vs 21 pregnant women without thyroid disease	12/22 women with previous PPT had recurrent disease. Half of these women had high thyroglobulin Ab or thyroperoxidase autoantibodies in the first trimester compared to none among those without recurrent PPT and 2/21 controls	Women with recurrent PPT were more likely to be smokers
Stagnaro-Green et al. (75)	Smoking cigarettes	4,394 women screened for thyroid function and TAb at 6 and 12 months postpartum	3.9%	No increased risk for PPT by smoking was found

Considering the relative rarity of the thyroid hormone resistance (RTH) syndrome, a non-autoimmune genetic disorder in which elevated circulating levels of thyroid hormones fail to suppress serum TSH, it is worthwhile mentioning two cases of association between RTH and PPT (70, 71) (Table 2). This association has been reported also outside of the postpartum setting (72). Concerning the role of cigarette smoking as a risk factor for PPT, data are controversial (73–75) (Table 2).

#### Postpartum Thyroid Hormone Autoantibodies

Positivity for TPOAb, already during the first trimester of gestation, is the best humoral marker for predictivity of PPT; nonetheless, only 33–50% of these women will develop PPT, whereas TAb-negative women have a very low incidence of PPT (53).

Some studies have reported the appearance of thyroid hormones autoantibodies (THAb) in postpartum period that can interfere with the evaluation of thyroid function. Jansson and Forberg described the presence of Ab against T3 in a woman who developed biphasic PPT, namely thyrotoxicosis followed by transient hypothyroidism after childbirth (76). In another study, three women with PPT had spuriously increased concentrations of free thyroid hormones because of antibody binding of radiolabeled T4 and T3 analogs. The antibody binding of radiolabeled analogs disappeared by 48 weeks postpartum. Postpartum women who develop THAb have about 2% prevalence of increased binding of radiolabeled analogs, which can result in an interference in thyroid hormone assays (77). Thus, it was suggested that women with PPT should receive full thyroid-function testing and be checked for possible antibody binding of analog tracers if free thyroid hormone levels are inappropriately increased (77).

Recently, the first case of transient appearance of THAb in pregnancy was reported (78). A 35-year-old pregnant woman, with a known diagnosis of HT, and under levothyroxine (L-T4) replacement therapy, was clinically euthyroid with normal TSH but elevated free triiodothyronine (FT3) and free thyroxine (FT4). Serum FT4 and FT3 returned progressively normal postpartum. The presence of circulating THAb was confirmed by demonstrating THAb against the two hormones (78).

In a Welsh larger study, THAb were searched, from week 4 through week 48 postpartum, in 148 thyroid antibody-positive women and 261 thyroid antibody-negative women (77). The study started with selecting, in the 409 women, those showing interference in the FT4 and/or FT3 assays using the corresponding free thyroid hormone Amerlex assay (77). Only 3 of the 148 women thyroid antibody-positive women (2.0%) had THAb, particularly T3-analog Ab (*n* = 1), T3-analog and T4-analog Ab (*n* = 2). Only two of the three women had PPT. Previously, the same group (79) found that the rate of THAb, as estimated by the interference (overestimation) of FT4 and/or FT3 by the Amerlex analog method, was 0.04% (1/2460), 50-fold less than the above 2.0%.

In these three women (77), THAb measured by non-specific precipitation of serum enriched with radiolabeled Amerlex-T4 analog or Amerlex T3-analog immunoglobulin classes, could not be identified. In the same three women (77), antibody binding of radiolabeled analogs and its effect (overestimation) on free T4 and free T3 assays disappeared by 48 weeks postpartum. However, there is the extreme variability of the THAb in causing interference.

In brief, one cannot predict the presence of THAb based simply on the interference (overestimation) in the assay of free thyroid hormones, since this interference is frequently absent. Furthermore, the greater the frequency of sampling and associated THAb search over a given time course, the greater the chances to detect THAb. Until now, there is no study that has evaluated their role as predictors for future development of PPT. However, it is noteworthy that THAb (at least one among T3IgM, T3IgG, T4IgM, and T4IgG) are highly prevalent in patients with DM1 (80), an autoimmune endocrine disorder that confers high risk for PPT (Table 2).

#### Clinical Picture

The clinical course of PPT is similar to subacute thyroiditis but in absence of anterior neck pain or tenderness of the thyroid (53). The classic form is characterized by transient hyperthyroidism (8–24 weeks postpartum), followed by a phase of transient hypothyroidism (3–12 months postpartum), and then by return to euthyroidism at the end of the initial postpartum year. However, the clinical course of PPT is variable because about 25% of women presenting the classical form, 25% isolated thyrotoxicosis, and one-half presenting isolated hypothyroidism (53). In the thyrotoxic phase, asthenia and irritability are the most frequent symptoms; instead, lack of energy, aches and pains, poor memory, dry skin, paresthesias, and cold intolerance are the most frequent hypothyroid features (81).

Approximately half of PPT women will have permanent hypothyroidism (53). Unless evolved into permanent hypothyroidism, PPT tends to recur after subsequent deliveries. A Welsh study (82) reported a 70% chance of developing recurrent PPT after the first PPT attack, and a 25% risk even in women who were only TPOAb positive without thyroid dysfunction during the first postpartum period.

#### Diagnosis

The diagnostic procedures depend on the phases of the disease. In the thyrotoxic phase (TSH suppressed with increased serum FT3 and FT4), it may be necessary to differentiate PPT from GD. In GD, radioactive iodine uptake is high, while it is low in PPT, but this test is contraindicated during breastfeeding. Therefore, in these cases, the dosage of thyroid receptor antibodies is useful: in PPT, they are absent in almost all cases. Also, the thyroid echography can to show an increased hypoechogenicity in many cases of PPT. In the hypothyroid phase, TSH levels are increased with low or normal FT4 concentrations (81). However, during postpartum, the susceptible period to develop GD and PPT is different: around 3 months for PPT vs later than 6 months for GD (83).

#### Nutraceuticals, Food, and Prevention Strategy of PPT

Selenium, through selenoproteins, has a key action on thyroid function by protecting thyrocytes from oxidative damage (84). Moreover, selenium supplementation can ameliorate thyroid autoimmunity in patients with mild-form or early-stage HT (85). Also, when given to pregnant women, selenium reduced serum levels of TPOAb and prevalence of PPTD and permanent hypothyroidism (85, 86).

Omega-3-fatty acid supplements can diminish the inflammation associated with certain autoimmune disorders, so that they could be used to treat AIT (43).

Concerning food, small oily fish consumption was reported to cure and/or prevent autoimmune disorders, and improve certain maternal and neonatal outcomes (87, 88). Small oily fish are a source of long-chain polyunsaturated *n*-3 fatty acids, while large predator fish contain a number of pollutants (87). Recently, in a study on pregnant women with stable dietary habits who were sampled throughout gestation and day 4 postpartum, fish consumers had lower rates of positivity of both TPOAb and thyroglobulin Ab compared to meat eaters. Furthermore, within the fish-eater women, those consuming small oily fish had lower rates of either Ab compared to those consuming large fish. Hence, pregnant women should avoid consuming swordfish and increase consumption of oily fish (87, 88).

#### Therapy and Follow-up of PPT

As appropriately underscore (53), “treatment of PPT is based on clinical experience because there have been no trials comparing therapeutic alternatives.”

In the thyrotoxic phase, if necessary, can be administered beta-blocker drugs, while antithyroid drugs are not recommended. During the hypothyroid phase, symptomatic women could be treated with L-T4 at replacement doses and L-T4 therapy is recommended in hypothyroid women who are attempting pregnancy or who are breastfeeding. If treatment is not initiated, TSH dosage should be repeated every 4–8 weeks until thyroid function normalizes. L-T4 therapy should be continued for 12 months (42).

## Author Contributions

FDB, RG, MLD, RV, and SB designed the study and reviewed the literature on postpartum and thyroid autoimmunity. SB and FDB wrote the manuscript. All the authors have revised the paper and approved the final edition.

## Conflict of Interest Statement

The authors declare that the research was conducted in the absence of any commercial or financial relationships that could be construed as a potential conflict of interest.

## References

[1] Wålinder O Addison disease during pregnancy – a diagnostic dilemma. Symptoms are similar to normal pregnancy problems. Lakartidningen (2005) 102:1988–90. 16044755

[2] Glazier MG Waldron WM. An unusual cause of postpartum vomiting. Arch Fam Med (2000) 9:284–6. 10.1001/archfami.9.3.284 10728117

[3] Xia Y Pan M Zhang Z Addison’s disease in pregnancy: a report of six cases. Zhonghua Fu Chan Ke Za Zhi (1996) 31:226–8. 8758779

[4] Drucker D Shumak S Angel A Schmidt’s syndrome presenting with intrauterine growth retardation and postpartum addisonian crisis. Am J Obstet Gynecol (1984) 149:229–30. 10.1016/0002-9378(84)90206-0 6720805

[5] Goldman MH Sfedu E Aboul-Hosn H Nutt R Addison’s disease presenting in the postpartum state. J Med Soc N J (1983) 80:1030–1. 6581317

[6] McGill IG Addison’s disease presenting as a crisis in the puerperium. Br Med J (1971) 2:566 10.1136/bmj.2.5761.566 5579195 PMC1795811

[7] Peschel G Addison’s disease in the puerperium. Z Gesamte Inn Med (1955) 10:810–4. 13282335

[8] De Bellis A Kelestimur F Sinisi AA Ruocco G Tirelli G Battaglia M Anti-hypothalamus and anti-pituitary antibodies may contribute to perpetuate the hypopituitarism in patients with Sheehan’s syndrome. Eur J Endocrinol (2008) 158:147–52. 10.1530/EJE-07-0647 18230820

[9] Watanobe H Kawabe H Painless thyroiditis developed in a patient with Sheehan’s syndrome. Painless thyroiditis developed in a patient with Sheehan’s syndrome. J Endocrinol Invest (1997) 20:335–7. 10.1007/BF03350313 9294780

[10] Takasu N Nakayama Y. A patient with postpartum hypopituitarism (Sheehan’s syndrome) developed postpartum autoimmune thyroiditis (transient thyrotoxicosis and hypothyroidism): a case report and review of the literature. J Thyroid Res (2011) 2011:413026. 10.4061/2011/413026 21603170 PMC3095901

[11] Sasaki H Ohnishi O Okudera T Okumura M Simultaneous occurrence of transient resolving thyrotoxicosis due to painless thyroiditis, hypopituitarism and diabetes insipidus following pituitary apoplexy. Postgrad Med J (1991) 67:75–7. 10.1136/pgmj.67.783.75 2057436 PMC2398922

[12] Zantour B Chadli-Chaieb M Maaroufi A Ach K Zaouali-Ajina M Tabka Z Post-partum autoimmune thyroiditis in a patient presenting with Sheehan’s syndrome. Ann Endocrinol (Paris) (2002) 63:223–5. 12193878

[13] Yoshida M Murakami M Ueda H Miyata M Takahashi N Oiso Y. An unusual case of hypopituitarism and transient thyrotoxicosis following asymptomatic pituitary apoplexy. Neuro Endocrinol Lett (2014) 35:342–6. 25275254

[14] Punnose J Agarwal MM Premchandran JS. Transient diabetes insipidus and hypopituitarism after pituitary apoplexy: a rare association with pericardial effusion and painless thyroiditis. Am J Med Sci (2000) 319:261–4. 10.1097/00000441-200004000-00012 10768614

[15] De Bellis A Colella C Bellastella G Lucci E Sinisi AA Bizzarro A Late primary autoimmune hypothyroidism in a patient with postdelivery autoimmune hypopituitarism associated with antibodies to growth hormone and prolactin-secreting cells. Thyroid (2013) 23:1037–41. 10.1089/thy.2012.0482 23286389

[16] Ertek S Erdogan G. Postpartum thyroiditis and hypothalamo-hypophysial insufficiency in the same woman with successive pregnancies: a case report. Gynecol Endocrinol (2010) 26:105–8. 10.3109/09513590903215532 19718560

[17] Bevan JS Othman S Lazarus JH Parkes AB Hall R. Reversible adrenocorticotropin deficiency due to probable autoimmune hypophysitis in a woman with postpartum thyroiditis. J Clin Endocrinol Metab (1992) 74:548–52. 10.1210/jcem.74.3.1310997 1310997

[18] Ozawa Y Shishiba Y Recovery from lymphocytic hypophysitis associated with painless thyroiditis: clinical implications of circulating antipituitary antibodies. Acta Endocrinol (Copenh) (1993) 128:493–8. 8393255 10.1530/acta.0.1280493

[19] Manetti L Parkes AB Lupi I Di Cianni G Bogazzi F Albertini S Serum pituitary antibodies in normal pregnancy and in patients with postpartum thyroiditis: a nested case-control study. Eur J Endocrinol (2008) 159:805–9. 10.1530/EJE-08-0617 18787047

[20] Landek-Salgado MA Gutenberg A Lupi I Kimura H Mariotti S Rose NR Pregnancy, postpartum autoimmune thyroiditis, and autoimmune hypophysitis: intimate relationships. Autoimmun Rev (2010) 9:153–7. 10.1016/j.autrev.2009.06.001 19539059 PMC2818005

[21] Sakinah SO Sharifah NA Yusoff K. Painful thyroiditis in postpartum period. Med J Malaysia (1993) 48:83–5. 8341177

[22] Akdemir Z Karaman E Akdeniz H Alptekin C Arslan H. Giant thyroid abscess related to postpartum *Brucella* infection. Case Rep Infect Dis (2015) 2015:646209. 10.1155/2015/646209 25861492 PMC4378610

[23] Hizawa K Okamura K Sato K Kuroda T Yoshinari M Ikenoue H Tuberculous thyroiditis and miliary tuberculosis manifested postpartum in a patient with thyroid carcinoma. Endocrinol Jpn (1990) 37:571–6. 10.1507/endocrj1954.37.571 2083534

[24] Carruthers DJ Peicher K Cabo Chan A Papillary thyroid carcinoma and subacute thyroiditis (Poster 223). Program & Meeting Abstracts, 86th Annual Meeting of the American Thyroid Association; 2016 Sept 21–25; Denver, Colorado (2016). p. 216, A-74.

[25] Park HK Kim DW Lee YJ Ha TK Kim DH Bae SK Suspicious sonographic and cytological findings in patients with subacute thyroiditis: two case reports. Diagn Cytopathol (2015) 43:399–402. 10.1002/dc.23230 25350937

[26] Ucan B Delibasi T Cakal E Arslan MS Bozkurt NC Demirci T Papillary thyroid cancer case masked by subacute thyroiditis. Arq Bras Endocrinol Metabol (2014) 58:851–4. 10.1590/0004-2730000003222 25465609

[27] Best CA Dhaliwal S Tam S Low TH Hughes B Fung K Spontaneous intrathyroidal hematoma causing airway obstruction: a case report. Medicine (Baltimore) (2016) 95:e3209. 10.1097/MD.0000000000003209 27583841 PMC5008525

[28] Davies TF. The thyroid immunology of the postpartum period. Thyroid (1999) 9:675–84. 10.1089/thy.1999.9.675 10447014

[29] Walfish PG Meyerson J Provias JP Vargas MT Papsin FR Prevalence and characteristics of post-partum thyroid dysfunction: results of a survey from Toronto, Canada. J Endocrinol Invest (1992) 15:265–72. 10.1007/BF03348726 1512416

[30] Gerstein HC. Incidence of postpartum thyroid dysfunction in patients with type I diabetes mellitus. Ann Intern Med (1993) 118:419–23. 10.7326/0003-4819-118-6-199303150-00004 8439115

[31] Shahbazian HB Sarvghadi F Azizi F. Prevalence and characteristics of postpartum thyroid dysfunction in Tehran. Eur J Endocrinol (2001) 145:397–401. 10.1530/eje.0.1450397 11580995

[32] Lucas A Pizarro E Granada ML Salinas I Sanmartí A. Postpartum thyroid dysfunction and postpartum depression: are they two linked disorders? Clin Endocrinol (Oxf) (2001) 55:809–14. 10.1046/j.1365-2265.2001.01421.x 11895224

[33] Hidaka Y Tamaki H Iwatani Y Tada H Mitsuda N Amino N. Prediction of post-partum Graves’ thyrotoxicosis by measurement of thyroid stimulating antibody in early pregnancy. Clin Endocrinol (Oxf) (1994) 41:15–20. 10.1111/j.1365-2265.1994.tb03778.x 7914152

[34] Rotondi M Pirali B Lodigiani S Bray S Leporati P Chytiris S The post partum period and the onset of Graves’ disease: an overestimated risk factor. Eur J Endocrinol (2008) 159:161–5. 10.1530/EJE-08-0236 18483014

[35] Rotondi M Cappelli C Pirali B Pirola I Magri F Fonte R The effect of pregnancy on subsequent relapse from Graves’ disease after a successful course of antithyroid drug therapy. J Clin Endocrinol Metab (2008) 93:3985–8. 10.1210/jc.2008-0966 18664537

[36] Jansson R Dahlberg PA Winsa B Meirik O Säfwenberg J Karlsson A The postpartum period constitutes an important risk for the development of clinical Graves’ disease in young women. Acta Endocrinol (Copenh) (1987) 116:321–5. 3687319 10.1530/acta.0.1160321

[37] Benhaim Rochester D Davies TF. Increased risk of Graves’ disease after pregnancy. Thyroid (2005) 15:1287–90. 10.1089/thy.2005.15.1287 16356094

[38] Tada H Hidaka Y Tsuruta E Kashiwai T Tamaki H Iwatani Y Prevalence of postpartum onset of disease within patients with Graves’ disease of child-bearing age. Endocr J (1994) 41:325–7. 10.1507/endocrj.41.325 7951587

[39] Ford HF Spies A. Postpartum proptosis with ophthalmopathy. Optom Vis Sci (2001) 78:75–8. 10.1097/00006324-200102000-00006 11265929

[40] Martin-du Pan RC Triggering role of emotional stress and childbirth. Unexpected occurrence of Graves’ disease compared to 96 cases of Hashimoto thyroiditis and 97 cases of thyroid nodules. Ann Endocrinol (Paris) (1998) 59:107–12. 9789594

[41] Nakagawa Y Mori K Hoshikawa S Yamamoto M Ito S Yoshida K. Postpartum recurrence of Graves’ hyperthyroidism can be prevented by the continuation of antithyroid drugs during pregnancy. Clin Endocrinol (Oxf) (2002) 57:467–71. 10.1046/j.1365-2265.2002.01615.x 12354128

[42] Alexander EK Pearce EN Brent GA Brown RS Chen H Dosiou C 2017 guidelines of the American thyroid association for the diagnosis and management of thyroid disease during pregnancy and the postpartum. Thyroid (2017) 27:315–89. 10.1089/thy.2016.0457 28056690

[43] Breese McCoy SJ. Coincidence of remission of postpartum Graves’ disease and use of omega-3 fatty acid supplements. Thyroid Res (2011) 4:16. 10.1186/1756-6614-4-16 22087511 PMC3231988

[44] Agard C Chaillous L Murat A Tranchant D Charbonnel B Postpartum thyroiditis and Basedow disease. Presse Med (1997) 26:1095–7. 9246101

[45] Nakamura S Isaji M Ishimori M. Thyroid hemiagenesis with postpartum silent thyroiditis. Intern Med (2004) 43:306–9. 10.2169/internalmedicine.43.306 15168773

[46] Shigemasa C Mitani Y Taniguchi S Ueta Y Urabe K Tanaka T Development of postpartum spontaneously resolving transient Graves’ hyperthyroidism followed immediately by transient hypothyroidism. J Intern Med (1990) 228:23–8. 10.1111/j.1365-2796.1990.tb00187.x 2384733

[47] Misaki T Miyamoto S Kasagi K Mori T Konishi J. Serial occurrence of two types of postpartum thyroid disorders. Usefulness of Tc-99m pertechnetate uptake. Clin Nucl Med (1996) 21:460–2. 10.1097/00003072-199606000-00005 8744180

[48] Papi G Corrado S Carapezzi C Corsello SM. Postpartum thyroiditis presenting as a cold nodule and evolving to Graves’ disease. Int J Clin Pract (2003) 57:556–8. 12918902

[49] Shorey S Badenhoop K Walfish PG. Graves’ hyperthyroidism after postpartum thyroiditis. Thyroid (1998) 8:1117–22. 10.1089/thy.1998.8.1117 9920367

[50] Momotani N Noh J Ishikawa N Ito K. Relationship between silent thyroiditis and recurrent Graves’ disease in the postpartum period. J Clin Endocrinol Metab (1994) 79:285–9. 10.1210/jcem.79.1.7913091 7913091

[51] Arpaci D Cuhaci N Saglam F Ersoy R Cakir B. Sheehan’s syndrome co-existing with Graves’ disease. Niger J Clin Pract (2014) 17:662–5. 10.4103/1119-3077.141447 25244283

[52] Le Donne M Settineri S Benvenga S. Early pospartum alexithymia and risk for depression: relationship with serum thyrotropin, free thyroid hormones and thyroid autoantibodies. Psychoneuroendocrinology (2012) 37:519–33. 10.1016/j.psyneuen.2011.08.001 22047958

[53] Stagnaro-Green A. Approach to the patient with postpartum thyroiditis. J Clin Endocrinol Metab (2012) 97:334–42. 10.1210/jc.2011-2576 22312089

[54] Dahale AB Chandra PS Sherine L Thippeswamy H Desai G Reddy D. Postpartum psychosis in a woman with Graves’ disease: a case report. Gen Hosp Psychiatry (2014) 36:761.e7–8. 10.1016/j.genhosppsych.2014.07.003 25194170

[55] Le Donne M Mento C Settineri S Antonelli A Benvenga S Postpartum mood disorders and thyroid autoimmunity. Front Endocrinol (2017) 8:91 10.3389/fendo.2017.00091 PMC5415609 28522989

[56] Amino N Mori H Iwatani Y Tanizawa O Kawashima M Tsuge I High prevalence of transient post-partum thyrotoxicosis and hypothyroidism. N Engl J Med (1982) 306:849–52. 10.1056/NEJM198204083061405 7062963

[57] Gudbjörnsson B Karlsson-Parra A Karlsson E Hällgren R Kämpe O. Clinical and laboratory features of Sjögren’s syndrome in young women with previous postpartum thyroiditis. J Rheumatol (1994) 21:215–9. 8182627

[58] van den Boogaard E Vissenberg R Land JA van Wely M van der Post JA Goddijn M Significance of (sub)clinical thyroid dysfunction and thyroid autoimmunity before conception and in early pregnancy: a systematic review. Hum Reprod Update (2011) 17:605–19. 10.1093/humupd/dmr024 21622978

[59] Friedrich N Schwarz S Thonack J John U Wallaschofski H Völzke H. Association between parity and autoimmune thyroiditis in a general female population. Autoimmunity (2008) 41:174–80. 10.1080/08916930701777629 18324487

[60] Amino N Tada H Hidaka Y. Postpartum autoimmune thyroid syndrome: a model of aggravation of autoimmune disease. Thyroid (1999) 9:705–13. 10.1089/thy.1999.9.705 10447018

[61] Stagnaro-Green A Akhter E Yim C Davies TF Magder L Petri M. Thyroid disease in pregnant women with systemic lupus erythematosus: increased preterm delivery. Lupus (2011) 20:690–9. 10.1177/0961203310394894 21436215

[62] Elefsiniotis IS Vezali E Pantazis KD Saroglou G Post-partum thyroiditis in women with chronic viral hepatitis. J Clin Virol (2008) 41:318–9. 10.1016/j.jcv.2007.12.010 18243783

[63] Bech K Høier-Madsen M Feldt-Rasmussen U Jensen BM Mølsted-Pedersen L Kuhl C Thyroid function and autoimmune manifestations in insulin-dependent diabetes mellitus during and after pregnancy. Acta Endocrinol (Copenh) (1991) 124:534–9. 2028711 10.1530/acta.0.1240534

[64] Gallas PR Stolk RP Bakker K Endert E Wiersinga WM. Thyroid dysfunction during pregnancy and in the first postpartum year in women with diabetes mellitus type 1. Eur J Endocrinol (2002) 147:443–51. 10.1530/eje.0.1470443 12370104

[65] Alvarez-Marfany M Roman SH Drexler AJ Robertson C Stagnaro-Green A. Long-term prospective study of postpartum thyroid dysfunction in women with insulin dependent diabetes mellitus. J Clin Endocrinol Metab (1994) 79:10–6. 10.1210/jcem.79.1.8027213 8027213

[66] Triggiani V Ciampolillo A Guastamacchia E Licchelli B Fanelli M Resta F Prospective study of post-partum thyroid immune dysfunctions in type 1 diabetic women and in a healthy control group living in a mild iodine deficient area. Immunopharmacol Immunotoxicol (2004) 26:215–24. 10.1081/IPH-120037717 15209357

[67] Jalkanen A Saraste M Gfeller A Surcel HM Airas L. Increased thyroid autoimmunity among women with multiple sclerosis in the postpartum setting. Mult Scler (2013) 19:1734–42. 10.1177/1352458513485148 23629943

[68] Maleki N Tavosi Z. Evaluation of thyroid dysfunction and autoimmunity in gestational diabetes mellitus and its relationship with postpartum thyroiditis. Diabet Med (2015) 32:206–12. 10.1111/dme.12580 25186500

[69] Komatsu M Kobayashi S Ito N Miyakawa M Senga O Sugenoya A A case of postpartum silent thyroiditis misapprehended as malignant lymphoma of the thyroid. Nihon Geka Gakkai Zasshi (1993) 94:645–7. 8341248

[70] Paragliola RM Concolino P De Rosa A Mello E Zuppi C Pontecorvi A The first case of association between postpartum thyroiditis and thyroid hormone resistance in an Italian patient showing a novel p.V283A THRB mutation. Thyroid (2013) 23:506–10. 10.1089/thy.2012.0080 23134553

[71] Taniyama M Otsuka F Tozaki T Ban Y Thyroid profiles in a patient with resistance to thyroid hormone and episodes of thyrotoxicosis, including repeated painless thyroiditis. Thyroid (2013) 23:898–901. 10.1089/thy.2012.0004 23240983

[72] Aksoy DY Gurlek A Ringkananont U Weiss RE Refetoff S. Resistance to thyroid hormone associated with autoimmune thyroid disease in a Turkish family. J Endocrinol Invest (2005) 28:379–83. 10.1007/BF03347207 15966514

[73] Galanti MR Cnattingius S Granath F Ekbom-Schnell A Ekbom A. Smoking and environmental iodine as risk factors for thyroiditis among parous women. Eur J Epidemiol (2007) 22:467–72. 10.1007/s10654-007-9142-1 17557139

[74] Balázs C Farid NR. Soluble CD4 concentrations predict relapse of post-partum thyroiditis. J Endocrinol Invest (2002) 25:11–7. 10.1007/BF03343955 11883861

[75] Stagnaro-Green A Schwartz A Gismondi R Tinelli A Mangieri T Negro R. High rate of persistent hypothyroidism in a large-scale prospective study of postpartum thyroiditis in southern Italy. J Clin Endocrinol Metab (2011) 96:652–7. 10.1210/jc.2010-1980 21190974

[76] Jansson R Forberg R. Influence of endogenous T3-autoantibodies on thyroid hormone measurements in a woman with transient postpartum thyroiditis. J Endocrinol Invest (1985) 8:253–6. 10.1007/BF03348488 4040937

[77] John R Othman S Parkes AB Lazarus JH Hall R. Interference in thyroid-function tests in postpartum thyroiditis. Clin Chem (1991) 37:1397–400. 1868601

[78] Teti C Nazzari E Galletti MR Mandolfino MG Pupo F Pesce G Unexpected elevated free thyroid hormones in pregnancy. Thyroid (2016) 26:1640–4. 10.1089/thy.2016.0112 27538922

[79] John R Henley R Shankland D. Concentrations of free thyroxin and free triiodothyronine in serum of patients with thyroxin- and triiodothyronine-binding autoantibodies. Clin Chem (1990) 36:470–3. 2311215

[80] Benvenga S Pintaudi B Vita R Di Vieste G Di Benedetto A. Serum thyroid hormone autoantibodies in type 1 diabetes mellitus. J Clin Endocrinol Metab (2015) 100:1870–8. 10.1210/jc.2014-3950 25710564

[81] Lazarus JH. Clinical manifestations of postpartum thyroid disease. Thyroid (1999) 9:685–9. 10.1089/thy.1999.9.685 10447015

[82] Lazarus JH Ammari F Oretti R Parkes AB Richards CJ Harris B. Clinical aspects of recurrent postpartum thyroiditis. Br J Gen Pract (1997) 47:305–8. 9219408 PMC1313006

[83] Ide A Amino N Kang S Yoshioka W Kudo T Nishihara E Differentiation of postpartum Graves’ thyrotoxicosis from postpartum destructive thyrotoxicosis using antithyrotropin receptor antibodies and thyroid blood flow. Thyroid (2014) 24:1027–31. 10.1089/thy.2013.0585 24400892

[84] Duntas LH Benvenga S. Selenium: an element for life. Endocrine (2015) 48:756–75. 10.1007/s12020-014-0477-6 25519493

[85] Wu Q Rayman MP Lv H Schomburg L Cui B Gao C Low population selenium status is associated with increased prevalence of thyroid disease. J Clin Endocrinol Metab (2015) 100:4037–47. 10.1210/jc.2015-2222 26305620

[86] Negro R Greco G Mangieri T Pezzarossa A Dazzi D Hassan H. The influence of selenium supplementation on postpartum thyroid status in pregnant women with thyroid peroxidase autoantibodies. J Clin Endocrinol Metab (2007) 92:1263–8. 10.1210/jc.2006-1821 17284630

[87] Benvenga S Vigo MT Metro D Granese R Vita R Le Donne M. Type of fish consumed and thyroid autoimmunity in pregnancy and postpartum. Endocrine (2016) 52:120–9. 10.1007/s12020-015-0698-3 26306774

[88] Le Donne M Alibrandi A Vita R Zanghì D Triolo O Benvenga S. Does eating oily fish improve gestational and neonatal outcomes? Findings from a Sicilian study. Women Birth (2016) 29:e50–7. 10.1016/j.wombi.2015.12.005 26837604

